# Looking Back and Looking Around: How Athletes, Parents and Coaches See Psychosocial Development in Adolescent Performance Sport

**DOI:** 10.3390/sports10040047

**Published:** 2022-03-22

**Authors:** Sergio Lara-Bercial, Jim McKenna

**Affiliations:** 1 Research Centre for Sport Coaching, Carnegie School of Sport, Leeds Beckett University, Leeds LS6 3QT, UK; j.mckenna@leedsbeckett.ac.uk; 2 Global Coaching Office, International Council for Coaching Excellence, Leeds Beckett University, Leeds LS6 3QT, UK

**Keywords:** positive youth development, youth sport, realist evaluation, life skills, personal development, psychosocial development

## Abstract

Sport has the potential to support psychosocial development in young people. However, extant studies have tended to evaluate purpose-built interventions, leaving regular organised sport relatively overlooked. Moreover, previous work has tended to concentrated on a narrow range of outcomes. To address these gaps, we conducted a season-long ethnography of a youth performance sport club based on a novel Realist Evaluation approach. We construed the club as a social intervention within a complex system of agents and structures. The results are published in this special issue as a two-part series. In this first paper, we detail the perceptions of former and current club parents, players and coaches, using them to build a set of programme theories. The resulting network of outcomes (i.e., self, emotional, social, moral and cognitive) and generative mechanisms (i.e., the attention factory, the greenhouse for growth, the personal boost and the real-life simulator), spanning across multiple contextual layers, provides a nuanced understanding of stakeholders’ views and experiences. This textured perspective of the multi-faceted process of development provides new insights for administrators, coaches and parents to maximise the developmental properties of youth sport, and signposts new avenues for research in this area.

## 1. Introduction

Over the last 40 years, sport has become integral to the lives of many children and young people (CYP). Beyond the typical array of physical and mental health and wellbeing benefits ascribed to participation (i.e., enhanced cardiovascular and musculoskeletal function and lower risk of obesity, diabetes and depression [1,2,3]), sport is also regularly presented as a tool to foster psychosocial development [4,5]. Given these potential benefits, governments and communities across the world continue to invest large amounts of financial and human capital in the promotion and provision of sport [6,7]. Whilst the physical and mental wellbeing benefits of participation have been studied extensively, a number of authors have contested the assumption that sport participation inevitably and automatically translates into psychosocial development [8,9]. Concerns have been raised that the extant literature fails to clearly define and fully understand the nature and extent of this phenomenon [8,10,11].

The study of sport as a tool for psychosocial development has been approached from three related yet distinctive perspectives, namely sport for development (SFD), life-skills development (LSD) and positive youth development (PYD). SFD is broadly defined as encompassing programmes that use ‘sport as a tool for development and peace’, aiming to maximise ‘the potential of sport as a tool to reach personal, community, national and international development objectives’ [12]. LSD typically focuses on the capacity for sport to teach youth the essential skills needed to successfully negotiate the demands of everyday life [13,14]. Finally, PYD has emerged as a broader field than LSD, focusing on the contribution that sport can make to foster positive and developmental behaviours and attitudes in young people, allowing them to thrive and transition successfully into young adulthood [15,16]. An in-depth review of this broad and extensive literature is beyond the scope of this paper (for this, see [17,18]). Nonetheless, a brief critique is presented to set the context for the current paper.

Research emerging from these three perspectives has enhanced our understanding of the properties of sport as a development agent. However, a number of concerns have been raised. First, there is no consensus regarding what constitutes personal development through sport [8]. Second, there is a tendency to study discrete outcomes using experimental designs to the detriment of establishing an integrated view of the process in natural settings [19]. Therefore, some authors have called for more process-based research focused on understanding the underpinning conditions and mechanisms that lead to positive psychosocial outcomes [13,20]. Finally, the field has been criticised for generally placing the focus on evaluating purpose-built interventions, thus overlooking regular organised sport in which most children take part [21]. As a result, some researchers suggest that many of the claims made for the psychosocial benefits of sport participation are only partially substantiated and lack ecological validity [8,22,23].

Youth sport researchers are thus being challenged to develop a deeper, systemic and integrated understanding of the factors and processes that lead to positive development [8,17,20,22]. To the best of our knowledge, a comprehensive exploration of sport-based psychosocial development meeting these criteria has not been conducted to date. The current study aims to address this gap in the literature through the ethnographic investigation of a youth performance sport club. In doing so, the new evidence base it produces will enhance the capacity of sport psychologists, coaches, parents, clubs and schools to foster positive growth. This evidence base will also be timely; it will contribute to providing essential political and financial support for youth sport to address the many harms accompanying the response to, and aftermath of, the COVID-19 pandemic.

## 2. Study Design and Methods

### 2.1. Theoretical Framework

To address the gaps identified above, this study adopted a novel realist evaluation (RE) approach [24,25]. RE typically aims to establish how well a given purpose-built social intervention achieves its expected outcomes. RE’s unique features have particular utility in the study of psychosocial development in organised youth sport when sport is construed and positioned as an organic social intervention embedded within a complex social system. In other words, environments where, with or without the awareness, intention and volition of those within it, a series of mechanisms interact, leading to explicit, implicit, desired and undesired developmental outcomes for all involved.

At an ontological and epistemological level, RE espouses a realist ontology and epistemology that accepts the existence of the social and material reality we interact with, yet states that the claims we can make about knowledge are always partial and never final. From this perspective, the role of the researcher is to constantly improve the existing available knowledge [24]. RE emphasises the fundamental role of theory in driving social research, and the importance of looking beyond quantification and correlation to shed light on the generative mechanisms affecting choices, behaviours and, ultimately, the outcomes of a given intervention. RE is thus concerned with the notion and nature of causality, focusing on “what works for whom, under what circumstances and why” [25] (p. 29).

Despite this focus on explanation and causality, RE accepts that social programmes take place within fluid and changing systems, meaning they feature flux, self-transformation and repatterning. Explained as ‘morphogenesis’ [26], RE implies that social interventions are never implemented in the same way twice, nor under similar conditions. Central to this variability is the intervention context. Given that social programmes are embedded into specific social systems, context is pivotal for how any programme works (or does not). From this perspective, programme outcomes emerge from a combination of agency and structure, the interface between the choices and capacities of contributing individuals and the collective resources at their disposal [24]. The fluidity and dynamism of the social world makes it challenging for the social evaluator to speak in absolutes or to make generalisations. Instead, RE recognises that certain elements and processes are relatively stable, and that these demi-regularities are the “subject matter of realist evaluation” [25] (p. 6). RE, therefore, subtly balances generalisability with specificity.

At a practical level, social programming and evaluation must start by making explicit the theories informing programme development and implementation, also known as programme theories (PTs) [24]. In RE, PTs comprise context–mechanism–outcome (CMO) configurations that allow researchers to make explicit not only the objectives of a programme (i.e., the expected outcomes of the intervention) within a specific context(s), but also the underlying assumptions about how a programme works (i.e., the generative mechanisms). As blueprints, PTs facilitate looking into the black box of programmes to establish not only if they work, but for whom, how and why. Having clearly articulated the PTs, researchers can explore how well these are realised in the “live” environment of the intervention; if they are not, or they are reached in unexpected ways, researchers can offer alternative explanations to create an “adaptive theory” [27], which is ever-evolving in light of new data [28].

Although RE has become a popular philosophical and methodological framework in social science in the last 30 years, this trend did not reach the sport literature until recently. RE-based approaches have started to proliferate in sport psychology [29], sport coaching [28,30], coach development [31], and sport policy [32]. Yet, it has not been used to explore young people’s personal development in and through sport. In this respect, RE not only allows, but also requires, researchers to integrate the wide number of ‘everyday’ factors that constitute ordinary sport club functioning into nuanced and fine-grained accounts of how psychosocial development may materialise. The resulting accounts will help sport psychologists, coaches and club administrators to become more effective, proactive and deliberate in their efforts to support it.

### 2.2. Study Design

To achieve the above, we conducted a case study investigation of psychosocial development in a youth performance basketball club in north-west England. Case studies entail a detailed examination of an individual unit, case or system, allowing researchers to achieve a deep understanding of their internal structure and operation [33]. By focusing on a single unit, case studies make it possible to address the complexity and individuality of social phenomena [34] in a live context [35]. In the context of previous research in this area, the case study approach allows researchers to address the need for reality, complexity and depth in the investigation of psychosocial development because the case being studied already exists—it is not manufactured for the purpose of the research [33].

Criterion-based sampling was used to select this setting [36]. The criteria included an environment featuring: (i) a high intensity and frequency of participant engagement combined with high future stakes (i.e., a professional career); (ii) a hybrid club ethos combining community- and performance-based values and provision, leading to a wide range of experiences and interactions; and (iii) an explicit humanistic philosophy combined with a prolonged high level of success at the national and international level. It is important to note the lead researcher’s familiarity with the setting, where he has coached for the last 20 years. The pros and cons of this element were carefully weighed regarding the balance of insider versus outsider positionality; the research team decided that the benefits outweighed the potential negative effects. Note also that none of the research subjects were or had ever been coaches coached by the lead author. On these bases, permission to progress with the study was provided by the club Chairman. Approval was subsequently granted by the Ethics Committee of Leeds Beckett University.

### 2.3. Participants

Purposeful sampling led to the identification of participants from three distinct samples: (i) former parents and players who had left the club at least 10 years ago; (ii) current parents and players; and (iii) current coaches. This aimed to generate two views of the same phenomenon. The former parents and players offered ‘updated yet retrospective views’. By contrast, the current parents and players provided ‘contemporary prospective views’ of their perception of day-to-day development, as well as anticipation of future benefits. The coaches, having worked at the club for multiple seasons, straddled a full range of these viewpoints. From an RE perspective, the depth and width of the accounts generated by such a group allows for a close understanding of the structure and impact of the whole programme by drawing on personal insights, reflecting different time frames of engagement and reflecting distinctive experience and expertise [1].

Five club coaches were selected (all male; mean age = 47; SD = 20.61). In addition, eight former parents (5 male and 3 female; mean age = 58.25; SD = 1.56), and six former players (all male; mean age = 30.83; SD = 1.77) were purposefully sampled based on their diverse trajectories after leaving the club (gaining a college scholarship in the US, turning pro, playing recreationally, etc.). Finally, 10 current parents (5 male and 5 female; mean age = 46,5; SD = 2.24) and 10 current athletes (all male; mean age = 13.9; SD = 1.57) were purposefully selected from the under 13 (U13) and under 16 (U16) teams (for full details of participants, see Table S1a–e in Supplementary Materials).

### 2.4. Data Collection Methods

A variety of data collection methods were used to elicit the PTs from the three stakeholder groups. Coaches and former parents and players were asked to take part in in-depth semi-structured interviews lasting an average of 70 minutes (range of 35 to 121). All interviews were tape-recorded and transcribed verbatim, producing over 350 pages of double-spaced text. The interviews commenced with a broad question: ‘In your view, beyond gaining physical and basketball-related skills, what is the impact of participation in the club programme at a personal level for the players?’. The interviewees were encouraged to elaborate on their responses, with examples, to explore different areas. Given the RE approach, the researcher explicitly probed interviewees to go beyond the identification of developmental outcomes and to articulate the how and why of these processes, asking questions like ‘Would you be able to explain to me how this happened?’ or ‘What factors do you think are responsible for this?’. The interviewees were also explicitly asked to consider the same issues from a negative standpoint (i.e., ‘From a personal development perspective, do you see any negative outcomes of being involved in the club?’).

By contrast, current parents’ and players’ PTs were identified through a series of focus groups (FGs). FG participants were selected working with the team coaches who were asked to identify a group of individuals with contrasting backgrounds and experiences to provide a greater variety of accounts (socioeconomic status, ethnicity, squad status, etc.). All in all, four separate FGs took place: two with U13 (*n* = 5) and U16 parents (*n* = 5), and two with U13 (*n* = 5) and U16 (*n* = 5) players. These lasted an average of 52.25 minutes (range 40–72). The FGs included similar questions to the in-depth interview, but in order to elicit as high a number of responses as possible, the participants were asked to initially write each developmental outcome on a sticky note and to submit all of their answers into an opaque container. Subsequently, the researcher took one item at a time out of the box and asked the participants to come forward to explain what they meant, or if no one came forward, asked the others if they had a similar experience. Throughout the FGs, the researcher encouraged elaboration to explore the features of the context and mechanisms that brought about specific outcomes. The FGs were tape-recorded and transcribed verbatim, producing over 60 pages of double-spaced text.

### 2.5. Data Analysis

**Figure 1:**
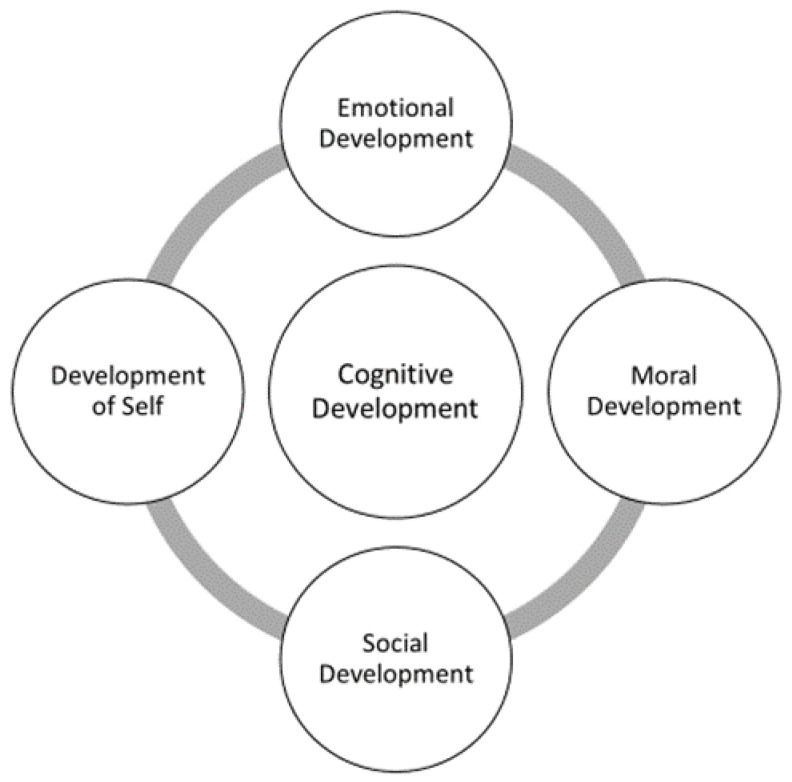
Psychosocial outcomes framework.

The interviews and FGs were analysed using a deductive–inductive iterative process facilitated by NVivo 10 software [37]. A three-stage process was implemented. First, the interview transcripts were read once to become familiarised with the content. Second, a deductive phase ensued in which the transcripts were read a second time, focusing on the identification of developmental outcomes. In line with RE tenets, this step was theory-driven and guided by a psychosocial outcomes framework (POF) created by the researchers through a substantial review of the developmental psychology literature (Figure 1; the full framework and review is available in the Supplementary Materials). Outcomes that did not fit into any of the categories of the framework were grouped as ‘Miscellaneous’. In the final, third stage, the analysis transitioned to an inductive approach focused on identifying generative mechanisms and their links to specific outcomes and to key contextual features. This process aimed to build a deep understanding of how coaches, parents and players construed the impact of participation in sport on psychosocial development.

## 3. Results

The study results are presented in four sections. The first two introduce the outcomes and generative mechanisms identified by all stakeholders using the POF as an organising tool. In the third section, the proposed mechanisms are explored in detail and a new practitioner-oriented categorisation, based on four broad families, is proposed. Finally, the fourth section details how the context modulated the impact of participation. For economy, given the broad range of findings, a combination of summative tables and text is used to introduce the findings, and interview and FG quotes are used sparingly. Full quotes can be found in Table S2a,b in the Supplementary Materials.

### 3.1. Participation-Based Outcomes

The analysis of the interviews with coaches, parents and players elicited a wide array of both positive and negative outcomes (Table 1).

A total of 21 positive and 11 negative outcomes were identified across the five development areas of the POF and an additional miscellaneous category. The social development category contained the highest number of positive outcomes (*n* = 6), whilst moral development contained the most negative outcomes (*n* = 4). With regard to single positive outcomes, a positive identity, a sense of hope and life purpose, a sense of belonging, a broader worldview, higher learning ability and work ethic were the most widely reported by interviewees. The below quotes illustrate these outcomes:

- Positive identity: “‘I’m pretty popular in my school. I even get away sometimes with stuff others don’t because I play in the basketball team.” (Kyle, current player)
- Sense of hope and life purpose: “This is what he wants to do, and this is the best possible place for him to do it in. He is determined to do it and he really feels like this place helps him get closer to his dream.” (Jerome, current parent)
- Sense of belonging: “Look, at the time, we lived in [name of town], it was a shithole, we couldn’t even go play outside and everyone was into football or rugby. Driving into [name of bigger city] to come to the centre was massive for me, having friends outside [name of town] was a salvation for me.” (Darren, former player)
- A broader worldview: “It was just great to see Sid interacting with all these different people. Where we live and where he goes to school most people are white middle class and he built some great relationships with kids that he would have never met otherwise, and I think that has stood him in great stead going forward to uni and now work.” (Mark, former parent)
- Higher learning ability: “It improves their concentration span. For him, it has gone through the roof, he has to pay attention to what coach is saying, to what’s going on around him, it really has helped him by being constantly exposed to information and coaching.” (James, current parent)
- Work ethic: “[…] sheer hard work, the boys learn how much harder they can work than they thought before. Coaches expect players to be on time, to do what they have to do, and to respect them and all around them. It builds a great work ethic.” (Coach George)

Conversely, low self-confidence, selfishness and uncontrolled aggression were the most discussed negative outcomes. The quotes below display examples of these components:

- Low self-confidence: “When they lose an important game or when they don’t play that much it’s hard, they are proper down and lose confidence. All you can do is comfort them and wait for them to bounce back.” (Sophie, current parent)
- Selfishness: “You always get two or three in a team that think they are the bee’s knees, and they become a problem.” (Coach Carl)
- Uncontrolled aggression: “I have said it before, they are a pack of alpha dogs trying to establish who the uber-alpha is going to be. They all come here having been the best in their local clubs, and now they have to work out who is top dog. And that can get hairy sometimes.” (Chloe, current parent)

### 3.2. Generative Mechanisms

Guided by the RE orientation of the study, identifying the generative mechanisms proposed by stakeholders was prioritised. Interviews elicited 68 mechanisms involved in the generation of the 21 positive outcomes (Table 2). Some of these were found to be more salient due to their involvement in the creation of more than one outcome: love for the game (*n* = 6), the inspirational coach (*n* = 6), success/winning (*n* = 5), playing other club roles (*n* = 5) and diversity (*n* = 4) were the most salient. Similarly, 16 mechanisms leading to negative outcomes were reported (Table 3). Internal competition (*n* = 5) and negative coaching behaviours (*n* = 3) were the most prevalent in the generation of multiple outcomes. Please note the addition of a miscellaneous category to account for outcomes that did not fit the POF.

### 3.3. Classifying Generative Mechanisms

Further to identifying the generative mechanisms and their associated outcomes, the mechanisms were grouped into related families. This exercise aimed to simplify the emerging complex picture and to start building a useable framework for practitioners. Four major groups of mechanisms were identified and named: (i) The Greenhouse for Growth; (ii) The Personal Boost; (iii) The Attention Factory; and iv) The Real Life Simulator. These families are defined below and presented in Table 4 and Table 5.

#### 3.3.1. Greenhouse for Growth

‘The Greenhouse for Growth’ relates to the built-in features of the setting that lay the foundation for the club to become a source of personal growth rather than just a sporting venue. Our ‘greenhouse’ allusion is intended to reflect generalised notions of a nurturing environment and climate. Four sub-categories were identified (i.e., club ethos, coaches’ behaviours, parental support/influence and social support influence), incorporating a further 17 mechanisms.

#### 3.3.2. Personal Boost

‘The Personal Boost’ focuses on the capacity of participation in performance sport to generate elevated states of mind (i.e., happiness, joy, satisfaction, elation, pride, etc.) leading to increased player wellbeing and a strengthened drive to stay involved in the sport and the club. Three main sub-categories were associated to this family (i.e., experience of success, athletic kudos, and steam release) containing six mechanisms.

#### 3.3.3. Attention Factory

‘The Attention Factory’ revolves around the notion of sport participation providing athletes with a clear focus in life that (i) leads to positive behaviours and outcomes and (ii) individuals use to confirm their personal agency. This attentional focus also acts as a protective shield and deterrent against negative sport-based attitudes and behaviours and insulates against engaging in rivalrous conducts seen (by most adults) as undesirable. Two sub-categories of mechanisms were created (i.e., love for the game and a purposeful life) encapsulating a further seven mechanisms.

#### 3.3.4. Real Life Simulator

Finally, ‘The Real Life Simulator’ relates to the idea that participation in youth performance development sport micro-replicates elements of the ‘adult world’. From this perspective, participation offers inexperienced young athletes multiple opportunities to practise facing and resolving challenges and situations that may recur in their future lives at university and/or in work. This family contains five sub-categories (i.e., competition, the team, learning, diversity and mini-workplace), encompassing 19 single mechanisms.

Table 4 and Table 5 summarise the mechanisms and sub-mechanisms in each major theme. This secondary analysis, aimed at increasing the practical application of these findings for coaches and club administrators, led to the further distillation of the mechanisms presented in Table 2 and Table 3, with some of them merging into a single theme for a new total of 49 mechanisms. A full depiction of the four families of mechanisms with sample quotes can be found in the Supplementary Materials.

### 3.4. Importance of Context

In keeping with the RE approach, the study also reflected on the impact of the specific context in which the club operated on the range of outcomes and mechanisms discussed by the contributors. Following the work of Pawson [25] (p. 37), we addressed four contextual layers: (i) individuals (i.e., the characteristics and capacities of the various stakeholders in the programme); (ii) interpersonal relationships (i.e., the stakeholder relationships that carry the programme); (iii) institutional settings (i.e., the rules, norms and customs local to the programme); and (iv) infrastructure (i.e., the wider social, economic and cultural setting of the programme). Individuals and their relationships will be treated jointly, as the interviewees reported them as inseparable.

#### 3.4.1. Individuals and Interpersonal Relationships

Four main groups of stakeholders were identified: club officials, coaches, parents and players. The club officials and coaches were primarily ex-high school teachers or past club players with a stated ‘genuine’ disposition to care for others, supplemented by years of successfully working with young people in school and sport settings. Their unity drove ‘what the club was about’: prioritising positive relationships and personal development over winning and external success. The parents typically felt this grouping of officials and coaches was key to the club operating as it did: this group provided the smooth alignment that integrated everyone at the club, while activating every player and every team to develop individually in positive ways.

Parents also played a significant part in the workings of the club. As a diverse cohort with a broad range of backgrounds (i.e., ethnic, educational and socioeconomic), parental involvement was typically and mainly seen as positive. Three profiles were described by the parents and coaches during the interviews and focus groups: (i) ‘peripheral parents’, who tended to stay outside of the activities and maintained a predominantly transactional relationship with the club; (ii) ‘rarely-seen parents’, who were typically working shifts, having to look after younger siblings or simply living too far away and having no transport; and (iii) ‘core parents’, who became club volunteers, playing roles such as team manager, mini-bus driver, table official, fund raiser, or match steward.

Parental dispositions reflected the levels and styles of engagement. Playing-based and player-based outcome expectations moderated this experience. Most parents recognised the slim chance of their son making a living out of basketball; they instead emphasised the ‘added value’ of participation as their main concern. For fewer parents, winning national titles, playing for Great Britain and getting a college scholarship were the main drivers for involvement. The ‘added value’ group deployed more adaptive and supportive attitudes; they focused more on the positive developmental outcomes of participation and less on their son’s playing time or performance. They were relatively content to ‘belong’ and to feel ‘included’ in the club environment. This attachment style may be moderated by the characteristics of the players, as described below.

The players, like their families, were also highly heterogeneous. Two main elements differentiated player experience and outcomes: (i) the level of parental support; and (ii) the pecking order within the club in terms of their expected progress. High parental support was linked to greater confidence, emotional wellbeing and emotional control. However, it was also connected to a sense of entitlement, or, in the case of overinvolved parents, to lowered confidence and outbursts of bad temper. Consistently ‘low’ parental support often created space for players to develop their resilience and self-responsibility, leading them to take charge of their own management and progress.

In relation to the playing pecking order, having high prospects and extended playing time was related to positive identity, a strong sense of hope and life purpose and higher confidence. Yet, a high ranking was also linked to taking a different interpersonal stance; selfishness and disrespect for lower-ranked players were noted. By contrast, players ranked lower risked developing low self-esteem, decreased emotional wellbeing and were prone to selfish acts to compensate. Despite this risk, powerful integrative and developmental daily routines (i.e., individualised training plans) coupled with a strong sense of identity meant that only few lower-ranked players exhibited these problematic responses.

#### 3.4.2. Institutional Settings

The institutional settings incorporate the rules, norms and customs local to the programme. Four main features were reported: (i) the explicit humanistic ethos of the club—rooted in the personal beliefs of the club founders; (ii) inclusivity—the club hosted both a national league programme and community leagues, and operated a low-cost policy to facilitate access; (iii) member expectations—all club members were expected to contribute to its smooth running by embodying its positive principles and carrying out the required chores; and (iv) the role of the basketball centre—the building itself created a socially rich environment that contributed to the development of a strong sense of belonging and enhanced wellbeing. As a result, the perception amongst all interviewees was that club members tended to spend much more time at the facility than was required by their training schedule. In effect, the basketball club seemed to operate as a *de facto* community centre where players and parents gathered to share in their love of the sport and each other’s company.

#### 3.4.3. Infrastructure

With regard to the wider social, economic and cultural setting in which the programme is embedded, a number of elements warrant special attention: (i) set in an inner-city neighbourhood of a large city in the north of England, the centre attracts a broad range of families from multiple ethnicities and socioeconomic backgrounds. Inner-city young people from lower income families shared the space with high income youths living in suburbs as far as 40 miles away. This diversity was seen by the interviewees as a catalyst for positive development, mirroring the real world and providing the children with a wider perspective and broader horizons; (ii) the northern location strongly translated into a sense of ‘northern identity’ characterised by grit, toughness and a need to prove oneself to ‘The South’; and (iii) the minority-sport nature of basketball in the UK underpinned the development of a marked ‘cult-like identity’. Belonging to a very unique ‘baller’ community, with a distinct lifestyle, leisure profile and sense of fashion, created a common identity. The low national profile of the sport with few professional opportunities also meant that the players and their families typically aspired to gain a scholarship to a USA college on completion of their secondary education. This facilitated a greater focus by the club on the long-term development of the players and on the prioritisation of academic achievement by players and their families.

In sum, the above description of the multiple layers of the context and their impact on the generative mechanisms and developmental outcomes serves to re-emphasise the integrated, multi-dimensional and highly individualised nature of the sport experience for young players. Table 6 provides a summary of the most salient contextual features.

## 4. Discussion

This study used an RE approach to study psychosocial development in youth sport in a novel way. This part one account has detailed the perceptions of former and current club parents, players and coaches, using them to build a set of programme theories: configurations of outcomes, context and mechanisms. The resulting network of outcomes and generative mechanisms spanning multiple contextual layers provides a nuanced understanding of stakeholders’ views and experiences. To respond to the many and dynamic needs of young people and the people who support them, the club appears to respond with a similarly complex offer, embodied by expectations and accountabilities built into its routines, practices and rituals. In line with the previous literature, no silver bullets for positive development were reported in this club [8,9]; instead, the high expectations placed on club members, and a group of stakeholders who were energised and committed to sacrifice and to hard work, done well and persistently, appeared to drive the outcomes [10,17,38]. Innovatively, this paper offers a textured perspective of the multi-faceted process of development. In doing so, it moves the study of psychosocial development in sport beyond the limitations of the existing research and identifies the depth and integration of what goes into creating developmental experiences in sport [19,20].

The results highlight the complex and systemic nature of the development process wherein a variety of factors and elements combine, interact and catalyse in multiple, non-linear ways to produce highly individualised outcomes. Within this context, and in line with previous research, the figure of the coach was revealed as a key modulator and catalyst [19,28,39]. Finally, in line with the previous literature, the study found that, despite a broad range of possible outcomes resulting from sport participation, the players’ internal and external assets and the personal narrative they attached to the experience all played a powerful role in determining the scale of their development [20,40].

Central to the novelty of this study, the use of an RE methodology addresses previous calls for using a more systemic and process-based research approach [13,19,20]. Identifying an emerging network of outcomes and generative mechanisms, filtered by individual, relational, institutional, and infrastructure contextual features, as hinted at by Kochanek and Erickson [41], this study moves beyond the typically reductionist approach taken in existing work. In doing so, it adds depth and practicality to current theoretical conceptualisations [13,20,42,43,44]. Likewise, this investigation answers the need to conduct ecologically valid research [21,23] by studying psychosocial development in a live organised youth sport setting, where the majority of the participants and practitioners operate. Moreover, this paper problematised development in youth sport by highlighting the potential for both positive and negative outcomes [8,9,22].

Most importantly, however, the strength of this study lies in highlighting the intricacy of youth sport as a developmental system. This is linked to the complexity of human development [45] and the myriad actors and potential generative mechanisms that combine—in multiple ways—to interact with the young person’s existing assets and personal narratives [38]. Unsurprisingly, this leads to highly individualised outcomes. Despite this inherent complexity, as called for in previous literature [21,23], using the RE framework has helped to make these intricate processes more accessible to practitioners [25]. By identifying and then classifying a clear set of developmental outcomes and mechanisms, sport psychologists, coaches and programme leaders are provided with a concrete menu of options to facilitate programme design and implementation. Similarly, the study highlights the return on investment from deploying a tailored, deliberate and integrative approach to personal development in organised sport [5,10]. This calls for all stakeholders to reconsider how their existing programmes address this wide range of features in their day-to-day processes.

Notwithstanding these findings, this paper represents only one half of the RE process. Once the stakeholder’s PTs are established ‘on paper’, it is necessary to test how these elements and processes feature in the ‘live’ environment. This ‘coalface’ analysis may reveal previously unseen components and relationships that challenge existing participants’ views; it may also unearth important new areas of inquiry and implementation. Layder refers to this process as the development of “adaptive theory” [27], the never-ending cycle of refining existing theory based on new emerging data to arrive at new insights. To address this, part two of the study, also published in this special issue, details the full-season ethnographic immersion of the lead author into the club’s environment. Through this multi-stage process, part two will also present an evidence-based, theory-driven and practitioner-oriented model of psychosocial development in youth sport, together with recommendations for future research.

## Figures and Tables

**Table 1:** Developmental outcomes of participation in youth performance sport.

Category	Positive Outcomes	Definition	Negative Outcomes	Definition
**Development of the Self**	Positive identity	A healthy view of oneself in the world	Quashed individuality Low self-confidence	Submission to the group’s needs above one’s own A reduced belief in one’s capability to deal with day-to-day issues
	Sense of hope and life purpose	A positive outlook of the future and that one’s life has meaning		
	Self-confidence	A belief in one’s capability to deal with day-to-day issues		
**Social Development**	Interpersonal skills	Competence to interact positively with others	Social isolation Selfishness	A sense of being cut off from friends and relatives Putting one’s personal needs and desires above those of the group
	Sense of belonging	A feeling of being part of something bigger than oneself		
	Social capital	A network of people around oneself which contributes to positive outcomes		
	Cooperation skills	Ability to work with others for a common purpose		
	Leadership attributes	Capacity to take initiative and to influence others		
	A broader worldview	An understanding of different perspectives and experiences		
**Emotional Development**	Emotional wellbeing	Overall high mental health	Low emotional wellbeing General demotivation	Reduced mental health Lack of motivation to do things beyond sport
	Emotional literacy	Understanding of a range of emotions		
	Emotional control	Being able to emotionally self-regulate		
**Cognitive Development**	Higher learning ability	Capacity to engage in learning opportunities	None recorded	
	Enhanced decision-making	Ability and proactivity to make decisions		
	Improved communication	Capacity to express oneself clearly and publicly		
**Moral Development**	Respect for others	Respecting others’ rights and feelings	Uncontrolled aggression	Bouts of aggression and hostility towards others
			Disrespect for others	Lack of respect for others’ rights and feelings
	Moral decision-making	Being able to tell right from wrong and decide accordingly	Bullying	Purposefully trying to hurt others physically or emotionally
			Occasional drinking	Episodes of drinking with teammates
**Miscellaneous**	Work ethic	Ability to work hard for long periods		
	Competitiveness	Desire to do well and succeed		
	Self-reliance	Capacity and proactivity to resolve problems independently		
	The springboard effect	Advantages later in life facilitated by their sport experiences		

**Table 2:** Generative mechanisms of positive outcomes of participation.

Development of Self	Social	Emotional	Cognitive	Moral	Miscellaneous
**Positive Identity** Success/winning Height as positive Being cool Love for the game **Sense of Hope and Life Purpose** Success/winning Inspirational coach Perception of progress Love for the game **Self-Confidence** Success and failure Competence Being cool Belonging to wider family	**Interpersonal Skills** Constant interaction Banter Social diversity Including new players Co-education Playing up an age group Positive use of social media Playing other club roles Coach as facilitator Interface with adults **Sense of belonging** Community spirit Belonging to wider family Soft hierarchy Common goals Looking out for others Love for the game Second home Sense of hope and life purpose Success/winning Inspirational coach All family involved High family time Family sacrifices Parent as volunteer One special friend **Social Capital** Belonging to wider family Diversity Playing up an age group Contact beyond basketball Positive use of social media **Cooperation Skills** Common goals Need to cooperate Understanding role and hierarchy From ‘I’ to ‘Us’ **Leadership Attributes** Being the captain Helping young players Doing the right thing **A Broader Worldview** Social diversity Travel opportunities	**Emotional Wellbeing** Caring environment Structure and routine The social network All family involved Letting off steam Competence Love for the game **Emotional Literacy** High exposure to range of emotions Inspirational coach Diverse responses to events **Emotional Control** Standards and expectations High pressure Coping with setbacks Putting team first Inspirational coach Parental support Parental presence Strong social network Steam release Being ready to learn and perform	**Higher Learning Ability** Constantly taught Coaching behaviours Constant feedback Setting personal goals **Enhanced Decision-Making Capacity** High-paced decision-making demands Love for the game **Improved Communication** Interface with adults Coach as role model Constant interaction	**Respect** Club values Putting team first Competition Being ready to learn and perform Playing other club roles Social diversity Skill diversity **Moral Decision Making** Club values Love for the game Competition Keeping busy	**Work Ethic** Club values Inspirational coach Internal competition Success/winning Family sacrifices Career ambitions Playing other club roles **Competitiveness** Inspirational coach High pressure **Self-Reliance** Standards and expectations Lack of parental support Career ambitions Playing other club roles **The Springboard Effect** Playing other club roles Strong social network Perceived added value of sport

**Table 3:** Generative mechanisms of negative outcomes of participation.

Self	Social	Emotional	Cognitive	Moral	Miscellaneous
**Quashed Individuality** Putting team first **Low Self-Confidence** Internal competition External competition Negative coach behaviours The parent coach	**Social Isolation** Lack of social time Being different in school **Selfishness** Burning desire to win Internal competition Parental behaviours	**Lowered Emotional Wellbeing** Pressure Lack of social time Lack of study time Negative coach behaviours Intra-team bullying **General Demotivation** Love for the game	None recorded	**Occasional Drinking** Steam release **Uncontrolled aggression** Internal competition External competition Parental behaviours Negative coach behaviours **Disrespect and Bullying** Daily internal competition Being different in school	None recorded

**Table 4:** Summary of generative mechanisms leading to positive outcomes by family.

The Greenhouse for Growth	The Personal Boost	The Attention Factory	The Real Life Simulator
**Club Ethos** Club humanistic philosophy and values High standards and expectations **Coaches’ Behaviours** Coach as inspiration Coach as facilitator Coach as role model **Parental Support/Influence** All family involved Family sacrifices Parental presence (or lack of) and contribution Proactive parental management by the club **Social Support/Influence** Soft hierarchy Community spirit and belonging Common goals Looking out for others One special friend The social network Second home Interface with adults	**Experience of Success** Exposure to success and failure Perception of progress and competence Sense of hope and purpose **Athletic Kudos** Being cool Height as positive **Steam release** Letting off steam	**Love for the Game** Personal infatuation with the game Collective infatuation with the game **A Purposeful Life** Career ambitions Structure and routine Sense of hope and purpose Short- and mid-term personal goals Keeping busy	**Competition** High pressure Coping with setbacks Internal and external competition Playing up **The Team** Putting team first Understanding roles and hierarchy Constant interaction Healthy banter Including players Looking out for others Do the right thing Chance to be a captain **Learning** Being constantly taught Constant feedback Setting personal goals High-pace decision-making **Diversity** Social diversity Geographical diversity **Mini-Workplace** Playing other club roles

**Table 5:** Summary of generative mechanisms leading to negative outcomes by family.

The Greenhouse for Growth	The Personal Boost	The Attention Factory	The Real Life Simulator
**Club Ethos** Putting team first (quashed individuality) **Coaches’ Behaviours** Negative teaching Negative emotions **Parental Support/Influence** Parental negative influence The overinvolved parent **Social Support/Influence** **Being different** Lack of social time Bullying	**Experience of Success** Exposure to success (feeling superior to others leading to disrespectful behaviours and a general sense of entitlement) **Steam Release** Letting off steam (leading to negative behaviours such as uncontrolled aggression or occasional drinking)	**Love for the Game** Personal infatuation with the game (leading to general demotivation to do anything other than sport)	**Competition** High pressure Pathological desire to win (leading to immoral decisions) Internal competition External competition

**Table 6:** Most salient contextual features.

Most Salient Contextual Features
**Individual and Interpersonal Level**
Personal values and beliefs
Socioeconomic status
Coaches’ professional background
Parental involvement and attitudes
Pecking order
**Institutional Level**
Club ethos
Club status
Club owned facility
High contact time
**Infrastructure**
Minority sport
Lack of professional pathways

## Supplementary Materials

The following are available online at https://www.mdpi.com/article/10.3390/sports10040047/s1, Table S1a–e: Participant demographic data. Table S2a,b: Generative mechanisms quotes examples.
