# Supplementary material for: Looking Back and Looking Around: How Athletes, Parents and Coaches See Psychosocial Development in Adolescent Performance Sport
Source: Sports (Basel). 2022 Mar 22;10(4):47. doi: 10.3390/sports10040047 (PMC9030748; doi:10.3390/sports10040047)
Supplement: Supplementary file 1 [file sports-10-00047-s001.zip › Supplementary Materials #1 - Participant Demographic Data Tables.pdf]

Table S1a. Demographic data of coaches.

Coach pseudonym	Age	Years coaching	Years at club	Former club player?	Current profession (or former if retired)	Socioeconomic status and ethnic origin
Jack	72	45	17	N/A Club co-founder	High school teacher	Retired middle class Professional White British
George	72	43	17	N/A Club co-founder	High school deputy head teacher	Retired middle class Professional White British
James	35	10	10	Yes	College teacher	Middle class Professional White British
Michael	26	6	4	No	Sport development officer	Working Class Semi-professional White British
Carl	30	8	8	Yes	Full time club coach	Working Class Semi-professional White British

Table S1b. Demographic data of former parents.

Former parent pseudonym	Age	No of children at club	Years at the club	Years children out of the club	Level children played at	Socioeconomic status; ethnic origin; marital status
Philippa and Jerry (interviewed together)	57 and 58	3	12	7	Junior and Senior National League	Upper middle class Professional White British/American Married
Albert	58	2	10	4	Junior and Senior National League	Upper middle class Professional White British Married
Damian	57	1	6	13	Junior and Senior National League	Lower middle class Semi-professional Black Caribbean Married
Mark	57	1	5	7	Top End Community League[1]	Upper middle class Professional White British Married
Patrick	58	2	12	7	Junior and Senior National League	Upper middle class Professional White British Married
Neil	59	2	5	11	Junior and Senior National League	Semi-skilled Working class Manual worker White British Married
Eva	62	1	7	6	Junior and Senior National League	Semi-skilled Working class Manual worker Black Caribbean Married (but husband still lives in Barbados)
Sandra	56	2	12	7	Junior and Senior National League	Semi-skilled Working class Manual worker White British Divorced

[1] Mark and his son Sid were chosen because, despite not reaching national league, Sid spent a high amount of time at the club. His experience as a 'near-miss' non-national league player was deemed valuable for the study.

Table S1c. Demographic data of former players.

Former player pseudonym	Age	Years at the club	Years out of the club	Level played at	Socioeconomic status; ethnic origin; marital status
Charlie	31	8	9	Junior and Senior National League<br>USA College<br>UK Pro League	Upper middle class Professional<br>White British/American<br>Single
Darren	32	6	7	Junior and Senior National League<br>UK Semi-Pro League	Lower middle class Professional<br>White British<br>Single (in long term relationship)<br>(currently lives in China)
Jacob	32	8	9	Junior and Senior National League<br>USA College<br>UK Semi-Pro League	Upper middle class Professional<br>White British<br>Married
Anthony	32	3	9	Junior and Senior National League<br>USA College<br>Europe Pro League	Upper middle class Professional<br>Black African<br>Married<br>(currently lives in the USA)
Sid	27	5	7	Top End Community League<br>UK University League	Upper middle class Professional<br>White British<br>Single
Steve	31	7	6	Junior and Senior National League<br>Europe Pro-League<br>UK Pro-League	Lower middle class Professional<br>Black Caribbean<br>Single (in long term relationship)<br>Has a daughter (6 years old)

Table S1d. Demographic data of current parents.

Current parent pseudonym	Age	No of children at club	Years at the club	Involvement at club beyond parenting	Socioeconomic status; ethnic origin; marital status
Jerome	48	3	5	Team manager<br>Mini-bus driver	Upper middle class<br>Professional<br>White British<br>Married
Thomas	49	1	4	None	Semi-skilled Working class<br>Manual worker<br>White British<br>Married
Will	46	1	5	None	Upper middle class<br>Professional<br>Black Caribbean<br>Married
Sam	45	1	3	Table official	Lower middle class<br>Semi-professional<br>White British<br>Married
James	48	2	4	Table official	Semi-skilled Working class<br>Manual worker<br>White British<br>Married
Emily	47	3	7	Team Manager<br>Table official	Upper middle class<br>Semi-professional<br>White British<br>Married
Chloe (Transgender)	45	1	3	None	Upper middle class<br>Professional<br>White British<br>Single (in long term relationship)
Megan	50	2	10	Team manager<br>Table official<br>General volunteering	Skilled Working class<br>Manual worker<br>White British<br>Divorced
Jessica	42	1	5	None	Lower middle class<br>Semi-professional<br>Black African<br>Married
Sophie	45	1	4	Team manager	Upper middle class<br>Professional<br>White British<br>Married

Table S1e. Demographic data of current players.

Current player pseudonym	Age	Years at the club	Playing history	Socioeconomic status; ethnic origin;
Harry	12	2	Played community basketball elsewhere<br>Joined national league programme 2 years ago	Upper middle class<br>White British
Oliver	13	1	Played community basketball elsewhere<br>Joined national league programme 1 years ago	Lower middle class<br>White British
Alfie	12	3	Came through club community programme<br>Selected to national league 3 years ago	Upper middle class<br>Mixed race White British/Caribbean
Jamie	13	3	Came through club community programme<br>Selected to national league 3 years ago	Working class<br>White British
Tabo	12	2	Played community basketball elsewhere<br>Joined national league programme 2 years ago	Working class<br>Black African
Ethan	15	4	Played community basketball elsewhere<br>Joined national league programme 4 years ago	Lower middle class<br>Mixed race White British/Caribbean
Noah	16	3	Played community basketball elsewhere<br>Joined national league programme 3 years ago	Upper middle class<br>White British
Mikael	15	2	Migrated from another country<br>Joined national league programme 2 years ago	Working class<br>Black African
Liam	16	4	Came through club community programme<br>Selected to national league 4 years ago	Working class<br>White British
Jayden	15	5	Came through club community programme<br>Selected to national league 5 years ago	Upper middle class<br>White British
