# Supplementary material for: Looking Back and Looking Around: How Athletes, Parents and Coaches See Psychosocial Development in Adolescent Performance Sport
Source: Sports (Basel). 2022 Mar 22;10(4):47. doi: 10.3390/sports10040047 (PMC9030748; doi:10.3390/sports10040047)
Supplement: Supplementary file 1 [file sports-10-00047-s001.zip › Supplementary Materials #2 - Mechanisms with Quotes.pdf]

Table S2a. Sample quotes of generative mechanisms leading to positive outcomes.

Family of Mechanisms	Sub-Family of Mechanisms	Definition	Sample Quotes
**The Greenhouse**	Club ethos	The club's humanistic philosophy and values paired with the continuous upholding of high-standards and expectations	*'The club has been the best thing that happened to them really. They have just been happy here. We have had ups and down, imagine, Stanley was diagnosed with Leukaemia, but the club, the coaches and the teammates helped us get through it all and here we are, stronger for it'* (Sandra, former parent)<br><br>*'It is part of the ethos of the club, we provide basketball opportunities for children and young people and their wellbeing is paramount'* (Jack, club founder)<br><br>*'If they do something wrong, there's a consequence and they never quite know what it's going to be. Each coach it's different, with each situation it's different and like in life, they then learn, it's like dropping a pebble in a puddle. There's reactions so they learn that.'* (Chole, current parent)<br><br>*'The kids know how to behave at the club, but because basketball matters so much to them, it is easier for them to manage their behaviour and emotions here than, let's say, in school'* (Thomas, current parent)<br><br>*'The doors are always open, people come and go as they please, it's more like a community club at times, players gather here and spend the evening here even if they are not training, just chatting to each other, watching others train or play.'* (Coach James)

			'I came on my own from a different country and everyone here took me under their wing, they fed me, they had me over for sleepovers, they even bought me a bible!.' (Anthony, former player)
	Coaches' behaviours	The coaches' positive influence mediated by their typical behaviours resulting in player inspiration, facilitation of development and positive modelling.	*'I look at people like [former teammate's name], for example, and you look at his situation outside of the club and you just think there's no way that kid should have... And it's nothing to do with his parents, single mum, all brothers in prison… that kid should not have been as well-mannered as he was. And you'd think, I genuinely think if it wasn't for basketball he wouldn't be like that. I think something would have called him in the wrong direction'* (Coach Carl)<br><br>*'I'm a single Mum and I've got two boys. I don't think I can teach them everything, so being in this environment with positive men around them makes them look at themselves and challenge themselves to do things differently or in a certain way.'* (Megan, current parent)<br><br>*'I think the coach's behaviour when you're in that losing situation, and when you've lost and when you've had a crap season and all that, how you handle that as a coach is going to influence the kids.'* (Patrick, former parent)<br><br>*'When two of them are not getting on well or there is tension or bad blood between them, the coach will sit them down and help them make up'* (Jerome, current parent).

		'Everyone wants to play for [name of club]. They all know this is the best club in the country, look at them banners on the wall. They win every year. They are really proud of that, and they want to be part of it." (Chloe, current parent)<br><br>*'The coaching, the style, the way they deal with things… certain coaches have better nurturing skills and are sympathetic, some of the more elderly experienced coaches. The kids that don't do well when they first join when they're a lot younger mentally suffer with that but when they get a bit of nurture, they absolutely shine. Alfie shone most of this year and I think he'll be able to handle a different coach this year.'* (Sophie, current mum)<br><br>*'The coaches are inspirational role models'* (Jerry and Philippa, former parents)
Parental support/influence	The parents' contribution to the experience of being in the club, including material and non-material contributions, player management and positive relationships with and within the club	*'Somebody asked my kids if I was an embarrassment because I go to all the games and I'm one of the loudest parents. And both my kids said it wouldn't be basketball without her, which I was really proud of because it wasn't forced and it was just said as a joke because we were laughing and joking about hearing me from one side of the court to the other.'*(Megan, current parent)<br><br>*'[we spent so much time together] because they were not old enough to travel, we were not local and we were in the car together 4 or 5 days a week for a couple of hours'.* (Albert, former parent)

			' [You have to] ensure parents understand what their role is and the best way to help their kids succeed; and that's not shouting at referees from the sideline or getting upset with them or coaching from the stands.' (Coach James).<br><br>*'The kids know they have to behave because their parents are watching. We don't want parents to interfere but they act as a deterrent.'* (Coach Carl)<br><br>*'I really like that basketball brings my family together, I feel like I really matter to my parents, and that's all down to basketball and the club.'* (Liam, current player)<br><br>*'When your son plays for the [club name], you also play, you are on call. We all do our bit to keep the club going, it is our club, it is for everyone's good.'* (Megan, current parent)
	Social support/influence	The stability and dependability afforded by the club's social structures including a soft hierarchy, a community spirit, common goals, special friendships, a shared and welcoming locale, and positive relationships with other adults.	*'You can just talk to any of these guys and they just listen because they're your friends and you've been playing basketball with them for ages'* (Liam, current player)<br><br>*'like family, you know, you see them [your teammates] every day and you do everything together'* (Noah, current player)<br><br>*'Yeah, definitely. I've not been in England for a long time and being here every single day helped me, the way I interact with others, get to learn new English*

			words every single day and understand better or my accent gets better, people understand me more. Before when I just used to go to school and not play basketball here, I wasn't even talking, I'd be quiet all day because I know if I say something, there's going to be someone there laughing at me because my accent is so terrible because I'm not from round here. Most of the time I'll just be there probably swearing at them in [name of foreign language] because they're laughing at me in English. But when I come here, these guys here respect me, they give me that confidence and that ability to talk. They helped me learn. That's really helped me a lot and I'm really grateful' (Mikael, current player)<br><br>*'We look out for each other; when I get upset someone will help me snap out of it; and I will do the same if I see someone lose it.'* (Mikael, current player)<br><br>*'For me it is the fact that there is always someone to talk to at the centre; I know that I can always come here and have a good natter with one of my mates and everything looks different after that.'* (Alfie, current player)<br><br>*'They stand out like a sore thumb on campus because they are comfortable talking to anyone, particularly adults like teachers because they have been doing that for ages with coaches.'* (Coach Carl)<br><br>*'You know, socially, it taught me, first of all, meeting and interacting with a lot of different people from a lot of different backgrounds. Some of those people,*

			they're still my best friends 15 years later.' (Charles, former player)<br><br>*'You have to be able to give it and also to take it. It goes both ways. It's never too mean, we can even talk about each other's mums and still control it. And it is just non-stop, we are at it all the time, even during training, and you know when someone has reached the end of their tether and then stop.'* (Liam, current player)<br><br>*'They are on it all the time, on facebook, on twitter, snapchat, through the playstation, never ending. They talk to each other, even when they are not together. I think it does help them with the relationship skills, it's constant practice, but also, they have to be careful and respectful of each other on social media, and we have never had any major issues there.'* (Emily, current parent)<br><br>*"Yeah, in here they build a relationship with other parents too, they get lifts with them, they have to talk to them and learn to get on with adults other than their own parents… or for instance, with the team manager and the centre staff.'* (Chloe, current parent)<br><br>*'The club was one big family, everyone knew each other and cared for each other. Parents would look out for everyone else's children.'* (Jacob, former parent)

			'Despite there being a clear hierarchy, the coaches, Jack the club director, it wasn't dictatorial, we all felt part of it, we all contributed to making decisions, it was everyone's club, not the coaches or Jack's only. The club belongs to everyone" (Charles, former player).<br><br>'*They all have that one teammate they are very close to. I think that's a big thing for them, they have this one person they really look forward to seeing every day, someone that whether the training or match goes good or bad is always there for them'.* (Coach James)<br><br>*'Players go out together all the time, they are in touch with each other constantly, yes, they still maintain a little bit their original circle of friends, but they also see the basketball guys outside basketball'* (Mark, former parent).
**The Personal Boost**	Experience of success	The regular exposure to instances of success as well as failure, a positive perception of progress and development, and the accompanying sense of hope and personal purpose	*'I think, initially, because you enjoy it, and also then obviously because you're good at it, because it does give you that confidence. I'm good at something, people look up to me. I think, yes, it's hard to say. When you're part of, say, a winning team, you have more confidence as a person and feel better'* (Neil, former parent)<br><br>*'Everyone wants to play for [name of club]. They all know this is the best club in the country, look at them banners on the wall. They win every year. They are really proud of that, and they want to be part of it."* (Chloe, current parent)

	Athletic kudos	The respect and admiration afforded by others for being a successful sportsperson	*'I'm pretty popular in my school. I even get away sometimes with stuff others don't because I play in the basketball team'.* (Kyle, current player)<br><br>*'I used to be very shy in school because of my height, until I started playing ball and then as we got older, I became a chick-magnet!'* (Alfie, current player)
	Steam release	The role of sport and physical activity as a release valve to defuse negative experiences outside sport	*'It calms me down when I'm angry with my brother. I come here and it calms me down. I used to be really angry when I was little and ever since I started playing basketball, I've been a lot calmer. It's just helped. It lets me let out energy, anger.'* (Thomas, current player)<br><br>*'You've just had a massive argument at home or something and you need to get away from the house, go to the gym or something, get some shots up, that's what you want to do because if basketball's your thing, that's going to get it off your mind, being able to go away and just shoot for a bit, work out. That's one thing basketball does, it relieves stress'* (Jayden, current player)<br><br>*'It provides an avenue for their energy and emotions to be channelled in a positive way, both in sport and outside. I think my son Harry behaves much better in school because he gets rid of all the negative energy at basketball.'* (Jessica, current parent)
**The Attention Factory**	Love for "The Game"	The personal and collective infatuation with the sport and its associated features and culture	*'[He is] consumed by basketball"* (James, current parent)

			'Basketball is all they can think about and all they want to do' (Chloe, current parent)<br><br>"*When basketball is off, they are a real sod, just don't know what to do with themselves, and I'm like, please can we start the season now?"* (Megan, current parent)<br><br>*'The kids know how to behave at the club, but because basketball matters so much to them, it is easier for them to manage their behaviour and emotions here than, let's say, in school'* (Thomas, current parent)<br><br>*'The mesmerising power of basketball; it's all he wants to do, it's all he wants to talk about, it's become his life day and night"* (Sophie, current parent).<br><br>*'The players are enthralled with the sport, and I think they love the fact that everyone here is too, we all think about it 24/7, it brings everyone together, it's nuts.'* (Coach Carl)
	A purposeful life	The existence of positive life goals coupled with positive expectations about them and daily routines to pursue them	*'It kept players focused and on the straight and narrow, and overall happier than if they were at home sat around the X-Box'* (Sandra, former parent)<br><br>*'It has helped, because then he's chosen that path of [sport], and he's been selective with his friends, the company he keeps. Because I think there's a lot of things that he didn't get into because of that being a physical sport, and keeping himself fit and clean, if you know what I mean' (Eva, former parent)*

			'If you've got a game on Saturday but you have a party on Friday night, don't go to the party because you've got a game on Saturday so you've got to get your priorities right… you want to make the right one and with discipline comes the right decision' (Harry, U13 squad)
**The Real Life Simulator**	Competition	Exposure to internal and external pressurised situations with potential for positive and negative outcomes and its consequences	*'Look, this is a winning club, we all have to understand that some kids play more than others, and that team selection is tough. And the kids get down on themselves sometime because of this, but it is part of the game. If they don't want to go through this, they should play community basketball not national league'* (Megan, current parent)<br><br>*'Well, really, if you can't take not playing or having to compete for minutes with your teammates, you probably shouldn't be in elite sport'* (Charles, former player)<br><br>*'There was only going to be one team, and then, Sam wasn't picked for that and he was gutted, he was crying before they actually made the announcement. Thank God they went with two teams and he made the second one'* (Sophie, current parent)<br><br>*'You can't let emotions get the best of you in games"* (Jayden, current player).<br><br>*'It is a sink or swim situation; they realise pretty early that if they are not able to keep a lid on their*

			emotions their game is going to suffer. The environment is not permissive of emotional outbursts.' (Coach James)<br><br>*'My Ethan can be down for days after losing a game, and all we can do is wait for him to come back to us. But it is normal, they have been working for this all year, and it is good to get over some disappointment… Tough, but they come out stronger at the other end"* (Megan, current parent)
	The Team	Working within a social, hierarchical structure such as a team where the greater good must be prioritised through the fulfilment of individual roles	['there was a very strong culture of] *putting the team always ahead of the individual"* (Thomas, current parent).<br><br>*'If you can't keep your cool, you are letting the team down.'* (Coach Michael)<br><br>*'Oh my, sometimes, when I have three or four of them together in the car going to a game, it's non-stop, they are on each other's face all the time in a nice way, they can give it and they can take it, and it never gets out of hand.'* (Jerome, current parent)<br><br>*'I loved the fact that we were all in the same boat, all trying to get the same objectives.'* (Anthony, former player)<br><br>*'Everyone is pulling together in the same direction, we are all in it together'* (Noah, current player)

		'This working as a team is one of the biggest things they get from being here.' (Coach George)<br><br>*'They need to understand where they stand in the team and get on with it, and do the best they can with the time they have for the benefit of the team. They have to let go of their egos.'* (Patrick, former parent)<br><br>*'Oh, I think... I don't necessarily think I was a leader before I came to the club. I think, because I'm eighth of ten kids, so I've gotten... I was bossed around from day one. But the coaches put me in a leadership position; expected me to go out there and play, and play hard enough that the rest of my teammates would follow suit.'* (Anthony, former player)
Learning	Exposure to constant teaching and feedback and setting of personal goals.	*'You cannot let your emotions get you down every time something doesn't go your way, because things go "tits up" all the time, you have to get up, dust yourself down and carry on'* (Jerome, current parent).<br><br>*'They need to be in a relatively calm state; you can't learn when you are all over the place or hyper. The kids realise that if they want to get better, they need to stay cool.'* (Coach James)
Diversity	The existence of a broad range of personalities and socioeconomic, cultural, and ethnic backgrounds	*'They are all so different and react to things in such a different way that this helps them understand emotions much better. The kids come from very different backgrounds and have very different coping mechanisms'* (Coach Carl)

		'You know, socially, it taught me, first of all, meeting and interacting with a lot of different people from a lot of different backgrounds. Some of those people, they're still my best friends 15 years later.' (Charles, former player)<br><br>*'You spend 4-5 hours in that crammed mini-bus and you end up talking with everyone and about everything. It is all about personal skills there. You may not like everyone, but you talk to them.'* (Jacob, former player)<br><br>*'Everyone has to get on with everyone else'* (Coach George)<br><br>*'It was just great to see Sid interacting with all these different people. Where we live and where he goes to school most people are white middle class and he built some great relationships with kids that he would have never met otherwise, and I think that has stood him in great stead going forward to uni and now work'* (Mark, former parent)<br><br>*'They are constantly mingling with the older groups and they pick a lot of things from that, they see what the next level of communication is, and also they talk to the younger ones and have to show them the way and be able to communicate with them too.'* (Megan, current parent)<br><br>*'Everyone is different, and you can't talk to them the same way, you have to find a way that works for every teammate.'* (Mikael, current players)

			'The girls are a great influence on the boys. They show them how to behave, and they are constantly talking to each other and chatting, and I think this helps the boys get better at talking to girls and not being "macho man" the whole time.' (Megan, current parent)<br><br>'*Through basketball they [the players] develop a very large network of people they can rely on, the other kids' parents, they make friends with the other kids' friends that otherwise they wouldn't have known, players from all over the country, their circle of friends really expands here'* (Damian, former parent)<br><br>*'The massive range of people he meets through basketball, not just the usual friends from school, but friends from all over the county, the centre is a meeting point for people from all over with one common interest, basketball.'* (Darren, former player)<br><br>'At their age, *most of their school mates have not been outside [northern city] unless they have gone for a holiday, somewhere, but these kids jump on a mini-bus every weekend and they travel to London, Newcastle, Birmingham, they are very lucky to be able to do this, I think it really helps them to understand the world is bigger than their school'.* (Coach George).
	Mini-Workplace	The replication of workplace-like situations within the club	*'When Ethan is refereeing on the adult league, that's a tough test. Grown men challenging his calls and he*

		boundaries fulfilling other roles beyond that of being a player	*has to stand his ground and not panic, and also not argue or be disrespectful.'* (Megan, current parent)

Table S2b. Sample quotes of generative mechanisms leading to negative outcomes.

**Family of Mechanisms**	**Sub-Family of Mechanisms**	**Definition**	**Sample Quotes**
**The Greenhouse**	Club ethos	The "club/team first" ethos resulting in a quashing of individuality	*'Even if sometimes we go overboard with this idea of working as a team, and it can become even detrimental to the development of superior talent, it is a cornerstone of the club, no-one is bigger than the club, they all have to muck in and work together for the greater good.'* (Coach Michael)<br><br>*'In this club, if they can't work for the greater good, they are going to last very little, we have seen it time and again before, selfish players get weeded out very quickly'* (Jessica, current parent).
	Coaches' behaviours	Negative coach behaviours in relation to some teaching approaches and emotion management	*'A particular coach was only guided by self-interest and this had a very bad impact on the kids at a mental level. It wasn't safe. Thankfully, this is rare and when it does happen it is rooted out very quickly because it goes against the club's philosophy.'* (Coach Carl)
	Parental support/influence	Negative impact of certain parental behaviours such as increased pressure or overinvolvement	*'[You have to] ensure parents understand what their role is and the best way to help their kids succeed; and that's not shouting at referees from the sideline or getting upset with them or coaching from the stands.'* (Coach James).

			'Some parents think their kid is God's gift to basketball and that gets passed on to the kid and they act like prima donnas.' (Sophie, current parent)<br><br>*'For the most part, it doesn't happen very often, and when it does we try to stamp on it early and strongly, we don't let parents coach from the sidelines or get too big headed about their own kids.'* (Coach Carl)
	Social support/influence	Isolation due to increased commitment to the sport or the lack of relevance of the sport in the school/neighbourhood environment	*'He spends most of his waking hours here training, he loves it, but at the same time it puts pressure on his other relationships, his friends from school…he can't hardly ever do anything with them because of basketball.* (Jerome, current parent)<br><br>*'The commitment required from basketball, I mean they are here three or four times during the week and then at the weekend both Saturday and Sunday, they haven't got time for anything else, their social life suffers'*. (Megan, current parent)<br><br>*'Of course they have to make sacrifices and they miss out on some things, but I think all they get from basketball makes up for it'.* (Coach James).<br><br>'*Being the weirdo that plays basketball because all the other kids were into football or rugby'* (Sam, Noah's dad).
**The Personal Boost**	Experience of success	Experience of success leading to a conceited attitude and a sense of	*'It is very difficult to know when normal banter and internal rivalry becomes bullying; it is difficult to*

		entitlement and lack of respect for others	*monitor and know exactly when to act, but overall, I think this is something that is stamped out pretty quickly when it happens”* (Coach Carl).<br><br>*‘Some of them really want to stand out and act in a bit of a selfish way, but slowly but surely they come round to the idea of team work.’* (Coach Carl)<br><br>*“You always get two or three in a team that think they are the bee’s knees, and they become a problem.”* (Jerome, current parent)<br><br>*‘[some of them are] so determined to make it to the next level that they became a little selfish and self-centred’* (Coach Michael)
	Steam release	Engagement in occasional aggressive or harmful behaviours.	
**The Attention Factory**	Love for “The Game”	Personal infatuation with the game leading to general demotivation to positively engage in other areas such as schoolwork	*‘The vast amount of time spent at the basketball club makes keeping up with school work and exams challenging and adds extra stress*.’ (Will, current parent)<br><br>*‘Basketball is all he wants to do. He can’t be bothered with anything else, school, mainly, comes second to basketball, and no matter what I say, he just wants to come here and play. I wish he was able to put the same amount of effort into school as he does for basketball.’* (Sophie, current mum)
**The Real Life Simulator**	Competition	Internal and external pressures which may lead to anti-social or immoral behaviours	*‘When they are down, they are down all right, it affects them a lot, and they bounce back quickly, but it is hard to see as a parent when your child is feeling like that.’* (Megan, current parent).

			‘There is a constant battle going on for selection. Some kids are definites, but my Alfie is in the middle of the pack, every weekend is a question mark, will he play, how many minutes will he get. He got stressed out over that a lot last year.’ (Sophie, current parent)<br><br>*‘My Ethan can be down for days after losing a game, and all we can do is wait for him to come back to us. But it is normal, they have been working for this all year, and it is good to get over some disappointment… Tough, but they come out stronger at the other end”* (Megan, current parent)<br>*‘They are constantly competing for spots for the weekend’s games, they have to battle it out on the court to make sure they get picked and that can get a bit hairy sometimes, it may make them very focused on their own goals and not the team’s’* (Chloe, current parent)
